# Mouthwash use and oral cancer: a systematic review and meta-analysis

**DOI:** 10.11606/s1518-8787.2023057004752

**Published:** 2023-11-08

**Authors:** Jennifer Sanzya Silva de Araújo, Elma Izze da Silva Magalhães, Hassan Lavalier de Oliveira Lima, Maria Carmen Fontoura Nogueira da Cruz, Erika Barbara Abreu Fonseca Thomaz

**Affiliations:** I Universidade Federal do Maranhão Departamento de Odontologia II Programa de Pós-graduação em Odontologia São Luís MA Brasil Universidade Federal do Maranhão. Departamento de Odontologia II. Programa de Pós-graduação em Odontologia. São Luís, MA, Brasil; II Universidade Federal do Maranhão Departamento de Saúde Pública Programa de Pós-graduação em Saúde Coletiva São Luís MA Brasil Universidade Federal do Maranhão. Departamento de Saúde Pública. Programa de Pós-graduação em Saúde Coletiva. São Luís, MA, Brasil; III Universidade Federal do Maranhão Departamento de Saúde Pública Programa de Pós-graduação em Odontologia São Luís MA Brasil Universidade Federal do Maranhão. Departamento de Saúde Pública. Programa de Pós-graduação em Odontologia. São Luís, MA, Brasil

**Keywords:** Mouthwashes, Mouth Neoplasms, Risk Factors, Meta-Analysis

## Abstract

**OBJECTIVE:**

This study aimed to investigate the effect of mouthwash use on the development of oral cancer.

**METHODS:**

Observational studies with adult/older adult populations that have examined the association between mouthwash use and oral cancer were included. Electronic search was performed in July 2022, with no time or language restrictions. PubMed/Medline, Embase, and Web of Science databases were used, and the search was extended to theses and dissertations libraries, Google Scholar, reference lists, and other sources. Methodological quality was assessed using the Newcastle-Ottawa Scale and quantitative data synthesis was performed by random effects meta-analysis, with different subgroup analyses and meta-regression. This revision was registered in Prospero (CRD42020143307).

**RESULTS:**

Of the 4,094 studies identified in the search, 15 case-control studies were included in the review, totaling 6,515 cases and 17,037 controls. The meta-analysis included 17 measures of effect from 15 case-control studies. The pooled OR was 1.00 (95%CI: 0.79–1.26, n = 17 studies), but it was 2.58 (95%CI: 1.38–4.82, n = 2 studies) among those who had used mouthwashes three times or more times a day, and 1.30 (95%CI: 1.10–1.54, n = 4 studies) among those who had used mouthwashes for more than 40 years.

**CONCLUSIONS:**

We found evidence that a high frequency of mouthwash use may be associated with an increased risk of oral cancer. However, despite the biological plausibility for this association, we suggest caution upon interpretation of our findings due to the few number of studies that have investigated the mouthwash use frequency, which should be considered. Therefore, we recommend that future studies assess, in detail, the frequency, duration, and content of mouthwashes to increase the strength of evidence for a possible dose-response effect of mouthwashes on oral cancer risk.

## INTRODUCTION

Oral cancer (OC) comprises tumors of the lip, oral cavity, and oropharynx^1^. It is considered a major public health problem worldwide^2^, being responsible for 476,125 new cases in 2020^3^. Squamous cell carcinoma represents more than 90% of this total^4^, commonly affecting men after the fifth decade of life^5^. OC is a complex and multifactorial etiology disease^5^, in which cells accumulate oncogenic stimuli and deviation from homeostatic mechanisms. Thus, a transition process from a normal to a dysplastic epithelium can be triggered by potentially malignant precursor disorders for the carcinoma^6^. Some of the major risk factors are tobacco use^7,8^, alcohol consumption^1,9^, age^10^, and sex^11^, as well as oral human papillomavirus infection, diet, genetics^12^, and persistent exposure to pathological or environmental cytotoxics^13^, without consensus about the mouthwashes use.

Mouthwashes have been used for centuries as breath fresheners, medicines, and antiseptics^14^ but the safety of their use and a likely association with OC have been widely discussed^15^. Different hypotheses have been investigated for the mechanisms involved in the carcinogenicity of alcohol-based mouthwashes, such as (1) intraoral oxidation of ethanol to its toxic metabolite acetaldehyde^17,22^, and (2) an accentuated local cytotoxic effect on human epithelial keratinocytes of the oral mucosa^13,23^. Cytotoxicity occurs when ethanol, in contact with the cells, induces deeper-layers stem cells to divide more often than normal to replace the damaged epithelium, leading to a variety of cancer-related errors, thereby increasing the risk of malignant transformation^23^.

The preponderant role of ethanol in the carcinogenic potential of alcoholic mouthwashes does not exclude the possibility that other components may also be involved in OC^13^. The impact of the complex mixture on oral cell’s cytotoxicity and antimicrobial activity is largely unknown^24^. Various molecules included in commercial mouthwashes are preparations created and proposed for the market^25^. In this way, it is possible that active antibacterial ingredients, other than ethanol, such as phenolic compounds^26^, triclosan^27,28^, cetylpyridinium chloride^29^, and chlorhexidine^30^ may increase the risk of OC by changing the diversity of oral bacteria^15^and causing cell damage^24^.

A previous systematic review^33^ and meta-analyses^34^ have investigated the association between mouthwash use and OC, but none of them found any evidence. The authors did not perform subgroup analyses considering adjusted and unadjusted estimates, type of controls, or frequency and duration of mouthwash use. Only Houstiuc et al.^34^ performed analyses in terms of duration and frequency of mouthwash use and alcohol content, but they only considered upper aerodigestive tract cancers, not OC.

In addition, although the searches have included the grey literature and reference lists, they were restricted to the main online databases, especially PubMed/ Medline, Web of Science, and Scopus. The PICO, PECO, or PEO strategies were not mentioned and few descriptors were inserted, and only studies published in English^34^ or English and Spanish^35^ were included. Furthermore, some of these meta-analyses^34,35^ included studies that may have contained overlapping samples^37^. This potential duplication occurred because these studies were part of multicenter research^44,45^ or were smaller in scale^37^. Moreover, the meta-analyses incorporated various types of studies, such as case series^46^, meta-analysis^36^, and studies focused on outcomes or objectives unrelated to oral cancer^17,47^. Therefore, since some studies indicate an association between mouthwash use and OC^15,44,45,55^, whereas other studies do not show such association, and considering the gaps left behind by previous meta-analyses, we propose to estimate the pooled effect of mouthwash use on OC depending on duration and frequency, type of control, and adjustment for confounding factors.

## METHODS

### Protocol and Registration

This systematic review with meta-analysis was reported following the recommendations of the Preferred Reporting Items for Systematic Reviews and Meta-Analyses (Prisma)^58^ and Meta-Analysis of Observational Studies in Epidemiology (MOOSE)^59^ guidelines. The detailed protocol (CRD42020143307) was registered in the International Prospective Register of Systematic Reviews (Prospero – Available at www.crd.york.ac.uk/PROSPERO).

### Context

This research aimed to answer the following questions: 1) Can mouthwash use be associated with OC? 2) Do mouthwashes have a dose-response relationship with OC? 3) How does the association behave depending on the alcohol content?

### Outcome

The primary outcome was the occurrence of OC (oral cavity and oropharynx) according to the International Classification of Diseases (ICD), 11^th^ Revision, 2B6E.0^60^. The anatomical subsites of the oral cavity consist of the labial mucosa, buccal mucosa, floor of the mouth, alveolar crest, gingiva, two anterior thirds of the tongue (anterior to the circumvented papillae), hard palate, and retromolar trigone, whereas the oropharynx consists of the soft palate, base (or posterior third) of the tongue, palatine tonsils, palatoglossal folds, epiglottic vallecula, and posterior pharyngeal wall^61^.

### Databases and Search Strategy

Systematic searches were performed in the following indexed databases: PubMed/Medline, Embase, Web of Science, Science Direct, Scopus (Elsevier), Biblioteca Brasileira de Odontologia (BBO), Dentistry and Oral Sciences Source - DOSS (EBSCO), Scientific Electronic Library Online (SciELO), LILACS, WHO Global Health Library, Directory of Open Access Journals – DOAJ, and Cumulative Index to Nursing and Allied Health Literature (CINAHL). Searches were also conducted using Google Scholar and grey literature from the Brazilian Digital Library of Theses and Dissertations. The reference lists of the included papers were also evaluated. The industries were contacted to request studies and data included in this study.

Initially two examiners were responsible for the search (JSSA, EBAFT). The PEO search strategy [Population (adults or older adults), Exposure (mouthwash use), and Outcome (OR)] was used. Thus, objective-related keywords, and MeSH terms (Medical Subject Headings) combined with Boolean operators (OR/AND/NOT) were used to ensure that the search strategy was comprehensive. The titles were searched in July 2022. Year of publication and language were unrestricted. The search strategy by database is detailed in Supplementary Table 1^a^. The searched study titles and their respective information were included in a Microsoft Excel^®^ 365 software spreadsheet (Microsoft Corporation, Washington, USA) to check for duplicity and to apply the eligibility criteria. Duplicate studies were excluded. The searches were compared, and any disagreement was resolved by the third reviewer (MCFNC).

### Eligibility Criteria

We included primary studies with adult or older adult populations that aimed to analyze the association between mouthwashes and OC. The excluded criteria included: 1) studies with specific populations with syndromes or congenital changes; 2) studies with more susceptible populations to the development cancer such as those previously exposed to chemotherapy or radiotherapy, and patients with specific genetic mutations; 3) publications involving the same population sample – in this case, the study with the major sample was selected; 4) studies with outcomes defined as dysplasia, cell damage, or nuclear alterations; and 5) letters to the editor, conference and congress abstracts, case series, case reports, in vitro studies, experimental studies in animals, review studies, and meta-analyses.

### Selection of Studies

An independent selection of studies was performed by two examiners (JSSA, EBAFT) and disagreements were resolved by consensus with the third reviewer (MCFNC). The first selection was based on the title and abstract, hiding the journal and author’s names, avoiding possible bias and conflicts of interest. Studies not selected at this stage or in the subsequent stages were registered in the spreadsheet as excluded, with their respective reasons. In cases where the study seemed to be eligible, but presented insufficient data in the title and abstract, the text was fully read and evaluated following the inclusion criteria afterwards. The full texts of the remaining studies were recovered and those eligible for this review were identified.

### Data Extraction

Relevant data from the selected articles were extracted, processed, and tabulated in a data collection form pre-developed in Microsoft Excel^®^ 365 (Microsoft Corporation, Washington, USA) by two reviewers (JSSA, EBAFT). All included articles were case-control studies, and the following data were recorded: authors of the studies, year of publication, country, recruitment period, sample size, age, gender (only one or two genders), type of exposure (mouthwash use – yes or no – and according to the frequency of use, alcohol content, and use duration over the years), type of outcome (OC site and ICD), type of controls (community or hospital), effect size (odds ratio), case-controls ratio, and variables considered in the adjustment for confounding (whether in pairing, sample restriction, or adjusted analysis).

For studies that reported measures of effect according to the cancer involvement site (oral cavity, pharynx, larynx, esophagus), those located in the *oral cavity* or *oral cavity and pharynx* were selected (when the measure of effect was simultaneously presented at both sites). For studies that reported effect sizes by categories regarding frequency of use or time of use, these measures were considered in subgroup analysis for dose response, sometimes being recategorized to allow comparability with other studies. For results stratified by gender, the measures of effect from each stratum were considered in the meta-analysis by inserting the letters *a* (men) and *b* (women). For studies that presented adjusted estimates for different confounding variable arrangements, the effect size adjusted for the largest number of variables was considered instead of potential mediators. Considering the possibility of residual confounding, subgroup analysis was performed considering three categories of adjustment: adjusted, when adjusted, at least, for age, gender, and tobacco and alcohol consumption; partially adjusted, when adjusted only for some of these variables; or unadjusted. Alcohol content could not be categorized, as information was missing in some studies, possibly because it was self-reported data. Data missing from the studies were disregarded. When the study did not present enough data to be included in the quantitative analysis, e-mails were sent to the authors to retrieve the data.

### Risk of Bias and Grading Quality of Evidence

The individual risk of bias of each study included in the systematic review was assessed by the Newcastle Ottawa Scale (NOS) for case-control studies by two independent examiners (JSSA, EISM). Differences were resolved by consensus in the presence of the third reviewer (MCFNC). The quality of evidence of the studies included in the meta-analysis was assessed following the GRADEpro Guideline Development Tool (GDT)^62,63^.

### Statistical Analysis

Stata 14.0 software (StataCorp, College Station, USA) was used for the meta-analysis. Since the heterogeneity evaluated by the I^2^test was high (77.1%), the DerSimonian-Laird Random-Effect method was chosen. Subgroup analyses were done with the studies that reported duration and frequency of mouthwash use to assess a likely dose-response, as well as to evaluate the subgroup according to the type of control (hospital or community) and the variables considered in the confounding adjustment.

Crude and multivariable meta-regressions were used to assess the contribution (%) of the co-variables [gender (men only; women only; and men and women), setting (low/middle-income or high-income country), sample size (up to 500; 501 to 1,000; and over 1,000 subjects), cancer site (only oral cavity or oral/pharyngeal/larynx sites), control type (hospital or community), and case-controls ratio (at least one case to two controls “1:2” or one case to one control “1:1”), OR adjustment] on the heterogeneity among the studies. Co-variables with p-value < 0.20 in crude meta-regression were included in the multivariable meta-regression. A funnel plot associated with the Egger regression asymmetry test was used to investigate the possibility of publication bias. OR were estimated and weighted by the study sample size and by their respective 95% confidence intervals (95%CI).

## RESULTS

### Searching Results

**Figure 1:**
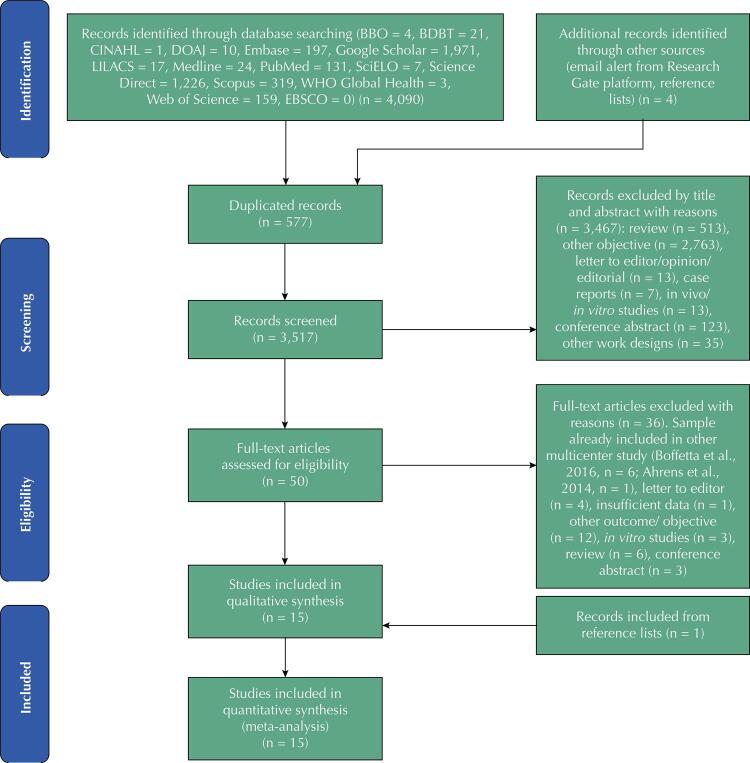
Study selection process evaluating the use of mouthwash and oral cancer.

Source: adapted from PRISMA Flow Diagram^51^.

We identified 4,094 records in the bibliographic search. After excluding duplicates, 3,517 titles and abstracts were read. Of these, 50 studies were selected for full-text reading, and 14 studies were included in our review, with one more paper identified after searching in the reference lists. Thus, 15 papers were included in the qualitative and quantitative analyses, totalizing 6,515 cases and 17,037 controls. The reasons for the 36 full-text articles excluded were: sample already included in other multicenter studies^44,45^ (n = 7); letter to editor (n = 4); insufficient data (n = 2); other outcome/ objective (n = 12); in vitro studies (n = 3); review (n = 5); conference abstract (n = 3) (Figure 1).

### Description of the Studies

Chart presents the main characteristics of the included studies. All studies featured the same design: case-control. Two studies were characterized as multicentric^44,45^ – one in different European countries (The ARCAGE study)^39^ and the other^45^ was a compilation of published and unpublished case-control studies in countries from America, Europe, and Asia. Other studies using these multicentric data^41^ were not included to avoid duplication of sample from the same survey. Studies were conducted in United States^15,64^, Brazil^57,69^, Italy^70^, China^71^, Australia^72^, Pakistan^56^, and India^55^. Most studies have used hospital controls and only three had community controls^15,45,67^. Seven studies had a proportion of at least two controls for each case^15,44,45,57,65,68,72^, and the others have used a 1:1 ratio. Six studies considered cancers in the oral cavity^15,45,55,57,71,72^, eight studies included oropharynx^44,64^, and one study also included the larynx as the outcome^56^.

**Chart:** Characteristics of the studies included in the systematic review and meta-analysis of the mouthwash use and oral cancer.

Study	Country	Recruitment period	Control setting	Sample size	Age range/median	Exposure (or exposure categories)	Outcome	Confounding variables considered in pairing, sample restriction or in regression analyzes	Risk of bias – NOS (Newcastle-Ottawa Scale)
Blot et al., 1983^57^	United States	1975–1978	Hospital	206 cases 352 controls	67	Years of mouthwash use (0-4, 5-9, 10-24, ≥ 25) and duration of retention in the mouth, frequency of use and concentration	Female patients with oral and pharyngeal cancer ICD, Rev. 8 (141,143-146,148,149)	Age, raçe, country, respondent (proxy or not) and tobacco habits	Low risk
Mashberg et al., 1985^58^	United States	1981–1983	Hospital	95 cases 913 controls	40-70+	Mouthwash use (people who used it routinely were those who reported using the mouthwash at least four times a week)	Oral and pharyngeal cancer	Tobacco habits	Moderate risk
Young et al., 1986^59^	United States	Unreported	Hospital	238 male cases 79 female cases 230 male controls 76 female controls	63.2/61.5	Mouthwash use	Oral cavity cancer, oropharyngeal cancer and hypopharyngeal cancer	Tobacco habits	Moderate risk
Winn et al., 1991^60^	United States	1984–1985	Community	573 male cases 293 female cases 821 male controls 428 female controls	18-79	Mouthwash use (people who used mouthwash at least once a week for six months or more). Initial age (years): <20, 20-29, 30-49, 50+, never. Duration (years): 0, 1-19, 20-39, 40+; Frequency (times / month): 0, 1-29, 30-59, 60+. Alcohol content: none, low ‘<25%’, high ‘≥ 25%’, mixed	Primary incidence of cancer of the oral cavity or pharynx ICD, Rev. 9 (141, 143-146, 148; 149)	Age, gender, race, education, study center, smoking, drinking, and fruit intake	Low risk
Talamini et al., 2000^61^	Italy	1996–2000	Hospital	132 cases 148 controls	27-86	Mouthwash use (times a week)	Cancer of the oral cavity and oropharynx	Gender, age, fruit and vegetable intake and smoking and drinking habits	Low risk
D’Souza et al., 2007^62^	United States	2000–2005	Hospital	100 cases 200 controls	≤ 50 ≥ 65	Mouthwash use (times a day)	Oropharyngeal SCC	Age and gender	Moderate risk
Marques et al., 2008^50^	Brazil	1998–2002	Hospital	309 cases (168 mouth cancer cases) 468 controls (406 mouth cancer controls)	40–70+	Mouthwash use (never, less than once a day, once or more times a day)	Mouth cancer and pharynx cancer ICD, Rev. 10 (C00- C06, C02.4 C05.1, C05.2, C09, C10)	Gender, age, schooling, smoking, alcohol consumption and all other oral health/hygiene variables	Low risk
Chang et al., 2013^63^	China	2010–2012	Hospital	317 cases (212 oral cancer cases) 296 controls	20-80	Mouthwash use (without alcohol or containing alcohol) or no use of mouthwash	Oral SCC located in the oral cavity, oropharynx, hypopharynx and larynx; people who has not been previously diagnosed with cancer	Gender, age, education, cigarette smoking (pack-year categories), betel quid chewing (pack-year categories) and alcohol intake (frequency)	Low risk
Eliot et al., 2013^19^	United States	2006–2011	Community	513 cases (143 oral cavity cases) 567 controls	56/ 60.5	Mouthwash use frequency (never; sometimes; at least once a day). Mouthwash use for alcoholic content (rarely or never; non-alcoholic; alcoholic). Rinse use by alcohol content and frequency (low or alcohol-free infrequently; Low or alcohol-free frequently; high with alcohol infrequently; high with alcohol frequently)	Incident cases of head and neck SCC ICD, Rev. 9 (141–146; 161)	Age, gender, race, smoking, alcohol consumption, education, annual household income, city, and history of periodontal disease	Low risk
Ahrens et al., 2014^39^	Multicentric (Spain, Czech Republic, Greece, Germany, Ireland, Italy, Norway, Croatia, United Kingdom)	2002–2005	Hospital	1 963 cases (925 mouth/oropharynx cancer cases) 1 981 controls	59.8	Mouthwash use frequency	ICD Rev. 10 (C00-06), oropharynx (C09, 10), hypopharynx (C12, 13), pharynx (C14), larynx (C32) or esophagus (C15)	Age, gender, study center, smoking status, a accumulative tobacco consumption, alcohol drinking duration accumulative alcohol consumption, SES/professional education, University degree, consumption of fruits and vegetables	Low risk
Alnuaimi et al., 2015^64^	Australia	2012–2013	Hospital	52 cases 104 controls	23-88	Regular alcohol mouthwash user	Oral SCC	Age, gender, and use of dental prothesis	Moderate risk
Assunção Junior, 2015^65^	Brazil	Unreported	Hospital	33 cases 20 controls	26-87	Mouthwash use, type of mouthwash and weekly frequency	SCC located in the oral cavity and oropharynx	Age and gender	Moderate risk
Boffetta et al., 2016^40^	Multicentric (United States, Puerto Rico, Argentina, Brazil and Cuba, Italy, Spain, Poland, Canada and India)	1981–2012	Hospital and Community	8 981 cases (2 790 oral cavity cancer cases) 10 090 controls (10 020 oral cavity cancer controls)	15-80	Mouthwash use. Duration of use in years: 0 (non-users), 1–15, 16–35 or 36+. Frequency of use per day: 0 (non-users), up to 1 time / day, more than once / day	Head and neck cancer ICD Rev. 10 (C00.3-C00.9, C02.0-C02.3,C03.0, C03.1,C03.9,C04.0, C04.1,C04.8,C04.9, C05.0,C06.0-C06.2, C06.8,C06.9;C01.9, C02.4,C05.1,C05.2, C09.0,C09.1,C09.8, C09.9,C10.0,C10.4, C10.8,C10.9;C12.9, C13.0–13.2, C13.8, C13.9;C32.0-C32.3, C32.8-C32.9	Study center, age, gender, cumulative tobacco smoking (pack-years), average amount of alcohol intake, and education level	Low risk
Saira et al., 2019^49^	Pakistan	2015–2016	Hospital	276 cases 275 controls	55/52.8	Mouthwash use	Carcinoma in the oral cavity, larynx, hypopharynx, oropharynx and pharynx	Age, race and language	Moderate risk
Sharma et al., 2020^48^	India	Unreported	Unreported	200 cases 200 controls	53.4/ 51.7	Mouthwash use daily	Oral SCC	Unadjusted for confounding bias	Moderate risk

SCC: squamous cell carcinoma; ICD: International Classification of Diseases and Health-Related Problems; Rev.: revision.

Except for Sharma et al.^55^, Mashberg et al.^58^, and Young et al.^59^, the other studies were matched minimally by gender and age. Other prevalent confounding variables included in multivariable analyses comprised tobacco and alcohol consumption, and, less often, fruit and vegetable consumption, ethnicity, socioeconomic conditions, among others. Human papillomavirus (HPV) was not included in the regression analyses of the identified studies. One study restricted the sample to people aged 40 years or older with no history of tobacco use^65^. One study provided only crude effects, i.e., no matching, no restriction, and no multivariable analyses^55^.

### Risk of Bias and Grading Quality of Evidence

According to NOS, eight studies presented a low risk^15,44,45,57,64,67,70,71^, and seven presented a moderate risk of bias^55,56,65,66,68,69,72^ (Chart; Supplementary Table 2^b^). In part, the methodological weakness of the investigated studies is their control selection since they present hospital controls. In addition, none of the studies reported the blinding of cases and controls in regarding exposure, which could have generated measurement bias.

### Meta-Analysis

Figure 2A shows the summarization of the 17 OR from the 15 studies included in the meta-analysis. Mouthwash use, regardless of alcohol content or frequency/duration of use, was not associated with OC (OR = 1.00; 95%CI: 0.79–1.26) and the heterogeneity among studies was substantial (I^2^: 77.1%). The funnel plot suggests a possible effect of the smaller studies, as they are more concentrated on the bottom right, but the Egger tests were not statistically significant (p = 0.651), indicating symmetry in the distribution of studies, and therefore a low possibility of publication bias (Figure 2B). When considering only the five effect estimates (OR) of the studies that analyzed alcohol-containing mouthwash *versus* no mouthwash use (Figure 2C), the overall weighed random effect increased but remained non-significant (OR = 1.20; 95%CI: 0.93–1.55).

**Figure 4:**
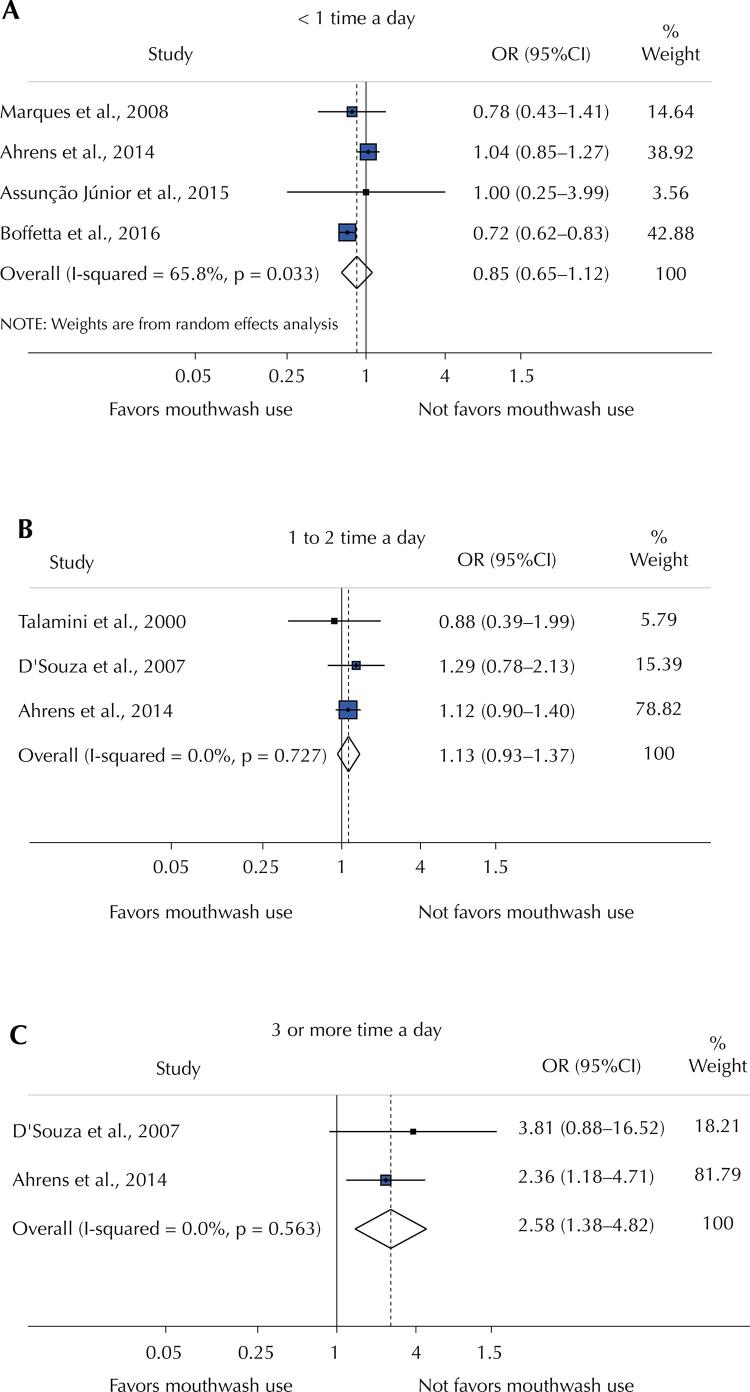
Meta-analysis of random effects of oral cancer odds ratio among mouthwash users and non-users considering the frequency of use < 1 time a day (A), 1 to 2 time a day (B) 3 or more time a day (C).

**Figure 2:**
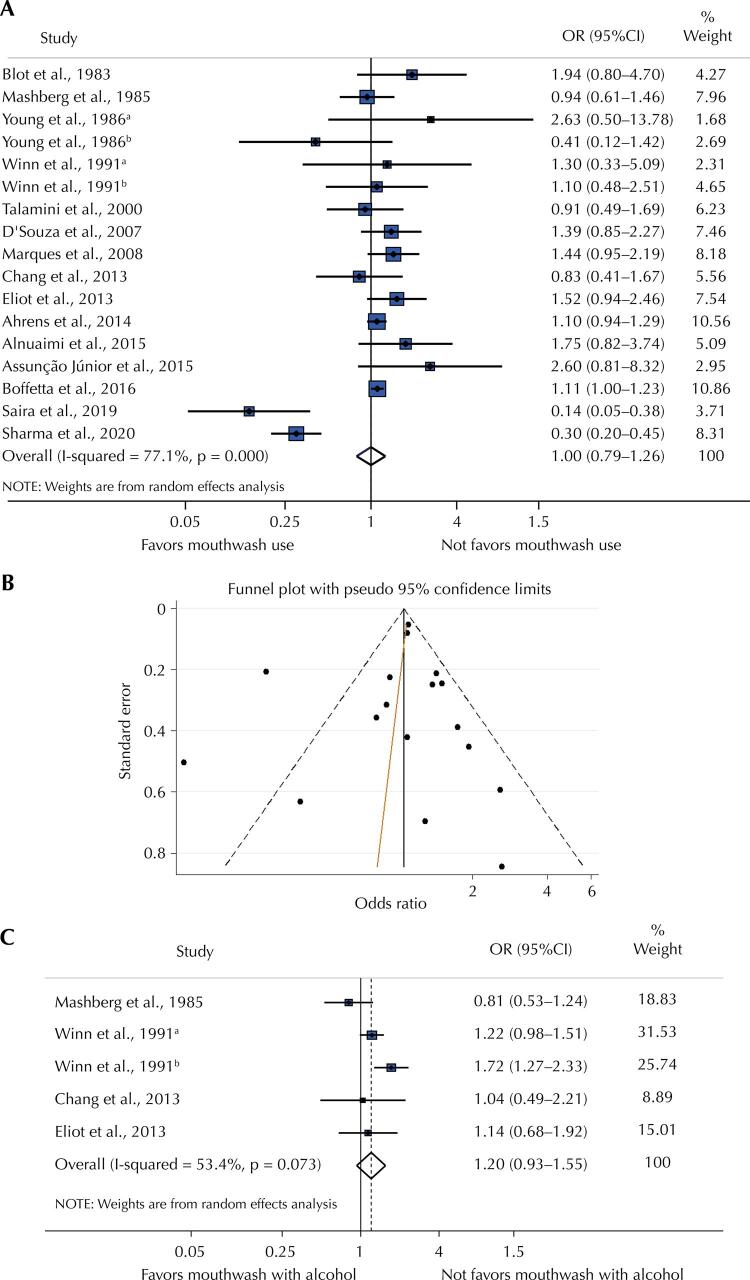
Meta-analysis of random effects of oral cancer.

OR: odds ratio; 95%CI: 95% confidence interval.

Note: (A) Meta-analysis of random effects of oral cancer odds ratio among mouthwash users versus non-users. (B) The funnel plot for this meta-analysis. The orange line represents the adjusted line corresponding to the Egger asymmetry regression test. (C) Meta-analysis of random effects of oral cancer odds ratio.

^a^ Gender male.

^b^ Gender female.

**Figure 3:**
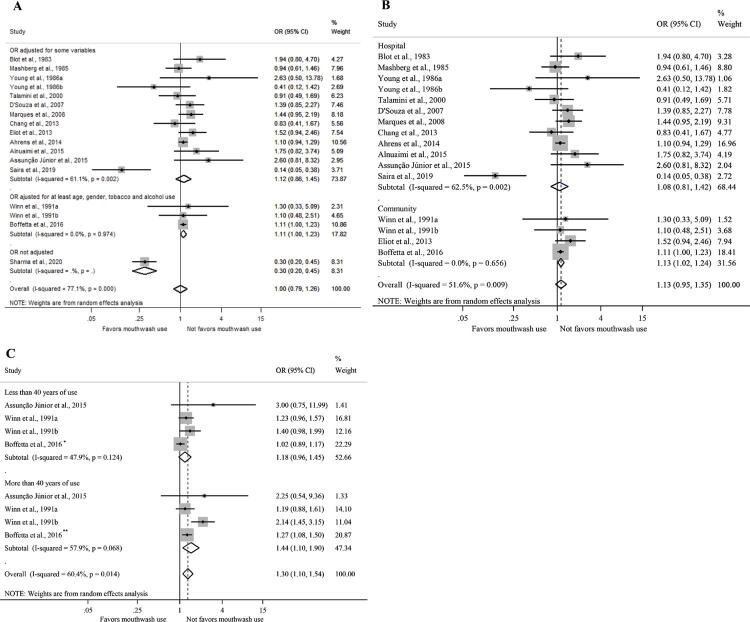
Meta-analysis of random effects of oral cancer odds ratio among mouthwash users and non-users, subgroup analysis according to the effect measure adjustment (A), control type (B) and usage time (C).

Subgroup analyses according to the subset of variables considered in the adjustment for confounding (OR = 1.00; 95%CI: 0.79–1.26) (Figure 3A) and control type (OR = 1.13; 95%CI: 0.95–1.35) (Figure 3B) did not show any association between mouthwash use and OC. However, when considering the frequency of use among mouthwash users (Figure 3C), the overall weighed random effect was significant (OR = 1.30; 95%CI: 1.10–1.54), showing that the use longer than 40 years was associated with 44% increased odds of OC compared with people who did not use mouthwash (OR = 1.44; 95%CI: 1.10–1.90).

OR: odds ratio; 95%CI: 95% confidence interval.

^a^ Gender male.

^b^ Gender female.

*Less than 36 years of use.

** More than 36 years of use.

Mouthwash use less than once a day (OR = 0.85; 95%CI: 0.65–1.12) (Figure 4A) and 1-2 times a day (OR = 1.13; 95%CI: 0.93–1.37) (Figure 4B), compared to no use, was not associated with OC. However, mouthwash use 3 or more times daily (Figure 4C) was associated with an increased chance of OC (OR = 2.58; 95%CI: 1.38–4.82).

### Meta-Regression

In the non-adjusted analysis, the co-variable ‘setting’ and ‘case-control ratio’ presented a p < 0.20 in association with OC, and explained 23.8% and 26.3% of the heterogeneity among the studies, respectively. Multivariable meta-regression showed that these variables, together, explained 39.4% of heterogeneity among the studies (Supplementary Table 3^d^).

## DISCUSSION

In this systematic review and meta-analysis of 15 case-controls and 17 OR estimates including 6,515 cases and 17,037 controls, we observed no association between mouthwash use (any *versus* no use) and OC (OR = 1.00; 95%CI: 0.79–1.26). Three previous meta-analyses also did not find association^34^. When Hostiuc et al.^34^ evaluated the overall risk of upper aerodigestive tract cancers associated with mouthwash use in 17 studies, the authors reported that the difference in risk between cases and controls was not significant. Argemi et al.^37^ also did not find association between mouthwash use and OC, neither when considered mouthwashes with alcohol in five case-control studies, nor without alcohol in four studies. Similarly, Gandini et al.^36^ estimated a non-significant relative risk summarized from nine studies. These authors also considered any frequency/duration of mouthwash use.

However, when we investigated the frequency of use, the odds of developing OC in individuals who frequently used mouthwashes (three or more times a day) was 1.30 times higher than in those who never used (OR = 1.30; 95%CI: 1.10–1.54); additionally, it was 158% higher among those who used mouthwashes for more than 40 years when compared to non-users (OR = 2.58; 95%CI: 1.38–4.82). This could suggest a dose-response effect. Gandini et al.^36^, however, estimated the relative risk with the frequency of use once, twice, or thrice a day and found no significant trend in risk with increasing daily use. Comparably, Hostiuc et al.^34^ found a non-statistically significant risk difference on the incidence of cancers in upper aerodigestive tract according to the frequency of use. We were not able to identify other meta-analyses that had assessed the dose-response effect related to OC.

Tobacco, alcohol, and betel consumption, diet, nutrition, as well as immunosuppression, environmental, and genetic factors are considered risk factors for OC^61,73^. When we performed subgroup analyses considering studies that reported both crude and adjusted associations, we reduced the probability that confounding biased the pooled estimates. However, the possibility of unmeasured confounding cannot be completely disregarded since important confounding factors could have been disregarded, such as HPV infection (not considered in any of the studies), tobacco and alcohol consumption, diet/nutrition, and socioeconomic conditions (considered only in some of the association estimates). Additionally, if a confounding factor is poorly measured or inadequately defined, residual confounding may also occur. However, we can suppose that the effect of the time of mouthwash use could be confounded by the age of the participants since increasing age is associated with increasing OC risk^10^. However, all studies included in this subgroup analysis have been adjusted for age and other potential confounders^45,67,69^.

Over the years, the main hypothesis for the link between mouthwashes and OC was the alcohol composition of these products. The carcinogenesis process would occur inducing a marked cytotoxic effect in human epithelial keratinocytes^13,23^, previously investigated *in vitro* with two commercially available mouthwash brands^14^. For each brand, an alcohol-free and an alcohol-containing version (96 mg/mL and 213.03 mg/mL, respectively) were tested on human oral keratinocytes with and without a mild dysplasia. The authors concluded that alcohol-based mouthwashes were genotoxic to both normal and dysplastic oral keratinocytes, inducing generalized changes in gene expression *in vitro*.

Similar results were also found in clinical trials evaluating the effect of alcohol-containing and alcohol-free mouthwashes on exfoliated oral cells^74,75^. In this context, the authors found an increased frequency of micronuclei and cellular abnormalities in the group exposed to the alcohol-containing mouthwash. Due to the superficial and intracellular characteristics of the oral mucosa epithelium, the detection of DNA damage and cell death in desquamated epithelial cells requires the genotoxic agent to overcome the permeability barrier of the basal layer and induce DNA damage, later converting them into micronuclei during cell division^76^. The correlation between the number of stem cell divisions that occurred in a tissue during a person’s life and the risk of cancer diagnosis in that tissue is highly positive and statistically significant^77^.

When considering only the use of alcohol containing mouthwashes *versus* no use, the association in our meta-analysis did not remain significant (OR = 1.20; 95%CI: 0.93–1.55). Argemí et al.^35^ also summarized data referred to the alcohol content of nine studies and showed a non-significant association (OR = 1.48; 95%CI: 0.85–2.56). Although the composition of mouthwashes and the alcohol content were not well described in all studies, the supposition that these non-alcoholic products with antimicrobial activity may also be cytotoxic should be mentioned^25,78^. A wide variety of antiseptics containing different active ingredients are available and widely used in dentistry^30^. These products are regulated as cosmetic products, thereby not requiring ingredients declaration^26^. Thus, we can assume that other components are also involved in cell damage^24^ or oral microflora alterations, harboring the potential to alter the balance of immune tolerance, further contributing to the genesis and promotion of OC^15^.

The most common molecules contained in mouthwashes are chlorhexidine, essential oils, cetylpyridinium chloride, triclosan, octenidine, delmopinol, polyvinylpyrrolidone, hyaluronic acid, and natural compunds^25^. When exposed to human gingival fibroblasts at the concentration required to inhibit 50% of cellular metabolic activity (IC50), 0.2% chlorhexidine decreased the viable cells number and increased the number of cells undergoing apopstosis^30^. Other *in vitro* studies^78^ corroborated these findings. Cetylpyridinium chloride was also found to exhibit severe cytotoxic effects against human keratinocytes and murine fibroblasts even at low concentrations^29^. Listerine^®^, a product that contains thymol, eucalyptus, methyl salicylate, and menthol, had its cytotoxicity evaluated^26^ and the authors have suggested all phenolic compounds may contribute, to some extent, to cell damage *in vitro*.

Triclosan is toxic to mitochondria, immune cells^27^, and possibly to the neural system^28^. In 2017, the Colgate-Palmolive company removed triclosan from dentifrices, following a determination by the United States Food and Drug Administration^82^. In addition to triclosan, twenty-three other active ingredients have also been removed from over-the-counter antiseptic products, due to insufficient data on their safety and effectiveness.

Hereupon, a limitation of our meta-analysis was the failure to perform subgroup analyses according to the different proportions of mouthwashes alcohol content. Otherwise, we could assess whether the substances present in their formulations are important for OC regardless of the alcohol content since the available evidence is supported only by *in vitro* studies. Thus, new studies that present data regarding the alcohol content of mouthwashes and their main components are essential to investigate and clarify the impact these molecules have.

This meta-analysis was also the first to analyze the quality of evidence using the GRADEpro GDT^62,63^. The tool estimated the quality of evidence as low. This result is mainly due to the design of the included studies. Case-control is the most feasible type of study design to investigate this subject, but it presents more biases than clinical trials and cohort studies. In this context, the possibility of some confounding, measurement, and selection biases leads us to classify the risk of bias as ‘serious’ by GRADEpro, despite most studies being classified as moderate or low risk of bias according to the NOS criteria. However, due to the unfeasibility of randomization, we can admit certain risk of bias in the case-control studies, so we can suggest that the NOS instrument, adopted in this meta-analysis, could have underestimated the risk of bias in the included studies. However, NOS is one of the most used instruments^83^, and its content validity and interobserver reliability are well established^83,84^. A recent meta-analysis on the topic^34^ did also use the same instrument; moreover, NOS seems to provide the same reliability, varying in applicability, compared to the ROBINS-I tool recommended by Cochrane. Furthermore, the complexity of using the ROBINS-I tool can be a limiting factor for its adoption^83^. Another factor that decreases the quality of the evidence is the inconsistency of results since some studies have showed positive (risk)^45,67^and others negative (protective)^55,56^ associations between mouthwash use and OC.

As strengths of our study, this meta-analysis was the first to consider the effect of the frequency and duration of mouthwash use over the years in OC. Despite pioneer, our findings should be carefully interpreted, given the small number of studies that considered the frequency (n = 2) and duration (n = 3) of mouthwash use. Another strength was the vast bibliographic search in a higher number of databases, including the grey literature, using the PEO strategy, without language and publication date restrictions. Therefore, we were able to reach studies that were not included in the previously published meta-analyses. In addition, we did not include, in this meta-analysis^37^, samples previously used in other larger studies^44,45^. We considered the alcohol content of mouthwashes *versus* the non-use when conducting the analyses, and different subgroup analyses were also performed. Lastly, a meta-regression was performed to explain the heterogeneity.

## CONCLUSIONS

This systematic review and meta-analysis showed no relationship between mouthwash use and OC, except for the mouthwash use for three or more times a day and for people who have used it for over 40 years, suggesting a possible dose-dependent effect. These findings, however, should be analyzed with caution given the small number of studies that consider the frequency of mouthwash use. Therefore, we recommend that future studies evaluate, in detail, the frequency, duration, and content of mouthwashes to increase the strength of evidence for a possible dose-response effect of this exposure on OC risk.

## Footnotes

a Available from: https://drive.google.com/file/d/1XhrSZK83w25gs21xPjPXbBngWiBQ8aXf/view?usp=sharing

b Available from: https://drive.google.com/file/d/10bjMWcTHo McovuRhrxkEzN9_eD2oGQLK/view?usp=sharing

d Available from: https://drive.google.com/file/d/1jFB_FOL8yj NnFgdEtuFgYFN3Ug8xeXsp/view?usp=sharing
